# Effect of PMMA Molecular Weight on Its Localization during Crystallization of PVDF in Their Blends

**DOI:** 10.3390/polym13234138

**Published:** 2021-11-27

**Authors:** Taotao Lin, Yongjin Li, Jiayao Wang, Jichun You

**Affiliations:** 1 College of Material, Chemistry and Chemical Engineering, Hangzhou Normal University, Hangzhou 310036, China; lintaotao1417@163.com (T.L.); yongjin_li@hznu.edu.cn (Y.L.); 2 Shanghai Institute of Applied Physics, Chinese Academy of Sciences, Shanghai 201800, China; 3 University of Chinese Academy of Sciences, Beijing 100049, China

**Keywords:** molecular weight, crystallization, exclusion, SAXS, DSC

## Abstract

In miscible crystalline/amorphous polymer blends, the exclusion behaviors of the latter with various molecular weights during the crystallization of the former were investigated by the combination of SAXS and DSC by taking a PVDF/PMMA blend as an example. The ratio between internal crystallinity from SAXS and overall crystallinity of the entire blend from DSC was employed to characterize the exclusion of PMMA. Our results indicate that the molecular weight of the amorphous component produces a remarkable influence on the diffusion coefficient (*D*) and the crystal growth rate (*G*) of the crystalline component. There are both inter-lamellar and inter-fibrillar structures when PVDF blended with lower-molecular-weight PMMA. With increasing molecular weight of PMMA, the decrease in crystal growth rate (*G*) dominates the enhanced exclusion behaviors of PMMA, resulting in bigger pores after extraction. Our results are significant not only for the basic understanding of crystallization in polymer blends, but also for the fabrication and structure control of porous structures based on crystallization templates.

## 1. Introduction

Miscible crystalline/amorphous polymer blends have attracted much attention because of their wide applications [1,2,3,4,5,6]. The amorphous component (i.e., diluent) can segregate in inter-lamellar, inter-fibrillar, or inter-spherulitic regimes [7,8,9,10]. As Keith and Padden suggested, the parameter *δ*, defined as the ratio between the diffusion coefficient (*D*) of the amorphous component and the crystal growth rate (*G*), plays an important role in the exclusion behaviors in polymer blends [11]. In the case of a higher growth rate (*G*) and lower diffusion coefficient (*D*), there is not enough time for the migration of the amorphous component, producing inter-lamellar structures. Conversely, the diluent will be excluded to the regions among lamellae stacks or spherulites when the ratio is high. According to this parameter, Yan et al. investigated the composition and isothermal temperature dependences of the exclusion behaviors in poly (butylene succinate)/poly (butylene adipate) (PBS/PBA) blend [12]. The results suggested that phase segregation of PBA takes place preferentially in the inter-spherulitic and inter-fibrillar regions at higher and lower temperatures, respectively.

In the investigation of the localization of amorphous components, considerable advances have been made by taking poly (vinylidene fluoride)/poly (methyl methacrylate) (PVDF/PMMA) as an example. PVDF/PMMA is a typical miscible crystalline/amorphous blend in the case of atactic- and syndiotactic-PMMA. The miscibility can be attributed to the favorable interactions between the carbonyl group of PMMA and the CH_2_ groups of PVDF [13,14]. The addition of PMMA leads to a lower melting temperature of PVDF and crystallinity [15,16,17]. The transition between inter-fibrillar and inter-spherulitic regimes has been achieved by means of composition and isothermal temperature, which has been validated with the help of various microscopes. For instance, Saito et al. investigated the coarse and compact spherulites in PVDF/PMMA using atomic force microscopy (AFM). Their results indicated that the former came from a two-step crystallization process while the growth rate was constant in the latter. In other words, the exclusion of PMMA played an important role in determining the morphology of the blend [18].

**Scheme 1:**
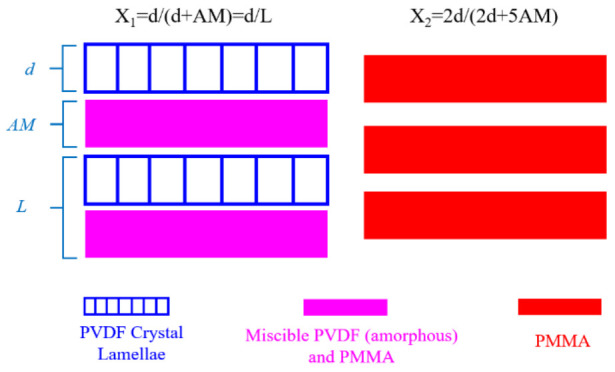
Schematic representation of *X*_1_ and *X*_2_.

Less attention has been paid to the inter-lamellar regime since its size is too small to track using microscopes. As a result, small-angle X-ray scattering (SAXS) has been introduced for the investigation of exclusion behaviors in lamellar scale [19,20]. To assess the segregation fraction of amorphous component in inter-lamellar and inter-fibrillar regions when they co-exist in the same system, Stühn et al. introduced an effective parameter by combining SAXS and differential scanning calorimetry (DSC) [21]. The internal crystallinity (*X*_1_) from SAXS is defined as the ratio between *d*/*L*, where *d* and *L* are lamellae thickness and the long period of the crystal, respectively; the overall crystallinity of the entire blend (*X*_2_, i.e., the mass ratio of crystallized PVDF over the blend, sic passim) from DSC can be calculated from the enthalpy values. The schematic representation of *X*_1_ and *X*_2_ is shown in Scheme 1. In brief, the crystallinity (*X*_1_) calculated based on the lamellae thickness and long period does not contain the excluded part of PMMA (red part in Scheme 1). The overall crystallinity (*X*_2_) considers the amorphous PMMA. However, it is impossible to evaluate the exclusion behaviors of PMMA based only on this parameter since the localization of PMMA is not clear. Hence, the ratio between *X*_1_ and *X*_2_ can act as an effective measure for the extent of PMMA exclusion. A higher value of *X*_1_/*X*_2_ corresponds to the enhanced exclusion behavior of PMMA. Using this parameter, Lin et al. clarified the role of molecular weight of crystalline component in determining the segregation morphologies [22]. The molecular weight of the amorphous component is expected to produce more profound effects on its exclusion behaviors because of the sensitive molecular weight dependence of growth rate (*G*) and diffusion coefficient (*D*) [23,24,25]. The basic understanding about this, however, remains obscure. In this work, therefore, PVDF/PMMA, a miscible blend that has been validated in reported results [18,20,24], was adopted as a model system to investigate the exclusion behaviors of PMMA, and its molecular weight dependences.

## 2. Experimental Section

### 2.1. Materials

PVDF (KF850, *M*_w_ = 209,000 g/mol, PDI = 2.0, ρ = 1820 kg/m^3^) was supplied by Kureha Chemicals (Tokyo, Japan). Four kinds of PMMA (CAS: 9011-14-7) with different molecular weights were purchased from Sigma-Aldrich (Saint Loius, MO, USA) and named PMMA-1, PMMA-2, PMMA-3, and PMMA-4 (listed in Table 1). N, N-dimethylformamide (DMF-99.5%, CAS: 68-12-2) and chloroform (CHCl_3_, AR) were provided by Lingfeng Chemical Reagent Co., Ltd. (Shanghai, China).

### 2.2. Preparation of PVDF/PMMA Blends

Before solution blending, PVDF and PMMA were dried in a vacuum oven at 80 °C overnight to remove moisture. PVDF and PMMA, with a weight ratio of 50/50, were mixed by DMF solution with a concentration of 10%. The solvents were evaporated in the vacuum oven under −0.1 MPa at 100 °C for 24 h. The PVDF/PMMA blends were then dried in a vacuum oven at 80 °C for 48 h to remove the solvent completely. After that, all samples were hot-pressed at 190 °C by 10 MPa for 5 min to obtain a membrane with a thickness of 300 μm. Finally, the membrane was melted at 200 °C for 30 min and then fully crystallized at 150 °C for 48 h. According to the reported results [18], crystallization at this temperature produces compact spherulites, which is a suitable model to investigate the enrichment of PMMA in inter-lamellar and inter-fibrillar regimes.

### 2.3. Dynamic Mechanical Analysis (DMA)

Dynamic mechanical analysis was performed with a DMA Q800 (TA Instrument, New Castle, PA, USA). All samples were cut into cuboid shapes (10 × 6.30 × 1 mm) and heated from −50 to 200 °C with a heating rate of 3 °C/min. A tensile mode with an amplitude of 10 μm and a frequency of 5 Hz was applied.

### 2.4. Differential Scanning Calorimeter (DSC)

The thermodynamic and crystallization behaviors of PVDF/PMMA blends were investigated by a DSC Q2000 (TA Instrument, New Castle, PA, USA) under nitrogen flow. Specimens were heated from 30 to 200 °C with a rate of 10 °C/min and held at 200 °C for 5 min to eliminate thermal history, and then cooled to 30 °C with a cooling rate of 3 °C/min. Before tests, the temperature and heat flow were calibrated with sapphire and pure indium, respectively. The glass transition temperature of PMMA was also evaluated by means of a DSC.

### 2.5. Polarized Optical Microscope (POM)

The crystallization dynamics of the PVDF/PMMA blends were investigated with the help of POM measurement (Olympus BX51, Tokyo, Japan). The POM was fitted with a charge coupling device (CCD) camera interfaced with a computer. The observed specimens were prepared by droplet drying from polymer blend solutions on glass sheets. A Linkam LTS 350 (Linkam, Tadworth, UK) hot stage was used to control the temperature. The samples were melted at 200 °C for 5 min to erase thermal history and then quickly cooled to 150 °C for isothermal crystallization of PVDF.

### 2.6. Small-Angle X-ray Scattering (SAXS)

Lamellar thickness, long spacing, thickness of the amorphous region, and crystallinity for the blends were calculated based on the information collected from a beamline BL16B1 (Shanghai Synchrotron Radiation Facility). The wavelength of the X-ray was 1.24 Å. The sample-to-detector distance was 1900 mm.

### 2.7. Thermogravimetric Analysis (TGA)

The removal of PMMA in blends was verified by thermogravimetric analysis (Q500, TA Instrument, New Castle, PA, USA). The temperature was raised from 30 to 650 °C at the rate of 10 °C/min in a nitrogen atmosphere.

### 2.8. Pore Size of Statistics

The samples were fractured in liquid nitrogen, and the fracture cross-section coated with gold was examined by field-emission scanning electron microscopy (FESEM, Hitachi S4800, Tokyo, Japan) at an accelerating voltage of 5 kV. Pore size statistics were carried out by a Nano Measurer (Department of Chemistry, Fudan University, Shanghai, China, software version: 1.2.5). Relevant data were obtained through the pore size statistics and analysis of porous PVDF phases in SEM images. The porous PVDF samples were prepared from the PVDF/PMMA blends after extraction with chloroform in a Soxhlet extraction device (Synthware, Beijing, China) for 72 h.

## 3. Results and Discussion

**Figure 1:**
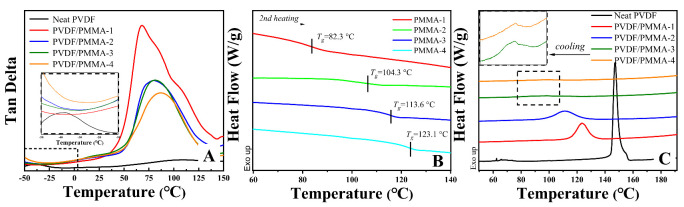
DMA (**A**) and DSC curves (**B**,**C**) of neat PMMA, neat PVDF, and PVDF/PMMA blends with different *M*_w_ of PMMA.

The miscibility between PVDF and PMMA with different molecular weights is the basis of discussing the effect on its exclusion behaviors. Here, the glass transition temperature (*T*_g_) of the polymer blend observed by DMA measurement was employed to evaluate the miscibility of two components. The dynamic loss for pure PVDF and PVDF/PMMA blends with different molecular weights of PMMA is shown in Figure 1A. Only one *T*_g_ was observed for pure PVDF, located at approximately −31 °C (shown in the inset in Figure 1A). All the PVDF/PMMA blends exhibited only one *T*_g_ (at 68, 79, 81, and 87 °C). The *T*_g_ of the blend increases with the increase in the molecular weight of PMMA [26,27,28]. As shown in Figure 1B, their glass transition temperatures were measured by means of DSC. With increasing molecular weight of PMMA, the glass transition temperature of PMMA increased significantly. The DMA results indicate that PVDF and PMMA are thermodynamically miscible. The crystallization behaviors of the PVDF/PMMA blends with different molecular weights of PMMA were investigated with the help of DSC. All samples were melted at 200 °C for 5 min to eliminate the thermal history completely and then cooled to 30 °C at a rate of 3 °C/min. As shown in Figure 1C, the exothermic peak of pure PVDF is located at 147 °C. After blending with PMMA, the crystallization temperature of PVDF in binary blends moved to a lower temperature. With increasing molecular weight of PMMA, the crystallization temperature decreased further (124, 111, 99, and 98 °C). The intensities of the exothermic peaks decreased significantly, indicating the lower magnitude of enthalpy during the non-isothermal crystallization process. Especially in their blends with PMMA-3 and PMMA-4, the crystallization peaks of PVDF almost disappeared. The reason for the dramatic decrease in crystallization temperature and enthalpy can be described as follows: On the one hand, with the increasing molecular weight of PMMA, the melt viscosities of blends increase. On the other hand, the longer chain of PMMA results in its stronger entanglement with PVDF, depressing the crystallization behaviors of PVDF [29,30,31]. This result is in agreement with the *T*_m_ depression effect from Nishi and Wang [32], which is discussed in the DSC heating curves in the following section. Both DMA and DSC results suggest the excellent miscibility between PMMA and PVDF [18,20,24,32].

**Figure 2:**
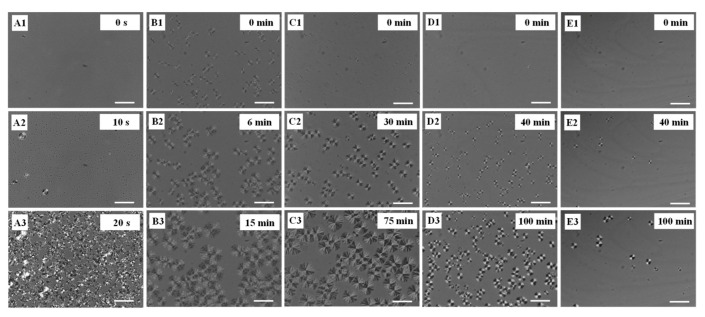
POM images of neat PVDF (**A1**–**A3**), PVDF/PMMA-1(**B1**–**B3**), PVDF/PMMA-2 (**C1**–**C3**), PVDF/PMMA-3 (**D1**–**D3**), and PVDF/PMMA-4 (**E1**–**E3**) isothermal crystallized for the indicated time at 150 °C. The scale bar is 50 μm.

**Figure 3:**
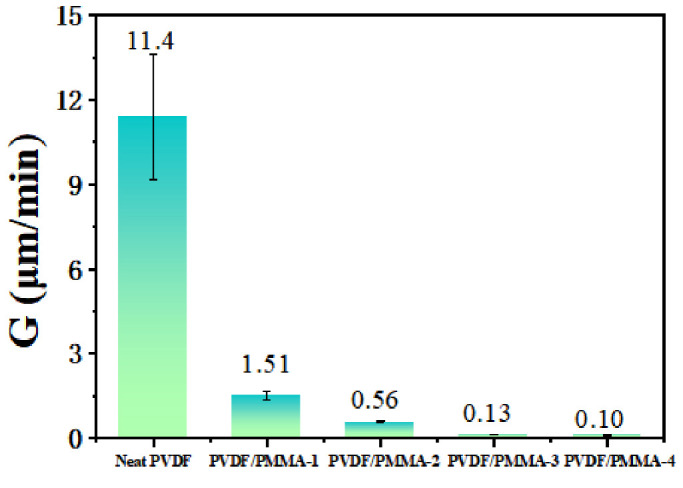
The growth rate of PVDF sphere diameter in neat PVDF and PVDF/PMMA blends crystallized isothermally at 150 °C with various molecular weights of PMMA.

The evolution of PVDF spherulites at 150 °C were tracked by means of POM (0 min was the starting point for the observation of spherulites). The spherulite growth of PVDF in PVDF/PMMA binary blends is shown in Figure 2. In all samples, the developing spherulites with a clear Maltese-cross pattern were obtained. According to these images, the growth rate (*G*) of PVDF spherulites diameter in blends with different molecular weights can be calculated based on the POM images. In the same way, it was easy to obtain magnitudes of *G* from all specimens, which are shown in Figure 3. The growth rate of neat PVDF is 11.4 ± 2.2 μm/min. The *G* value in binary blends gradually decreased from 1.51 ± 0.18 to 0.10 ± 0.01 μm/min upon increasing molecular weight of PMMA, which can be attributed to the higher viscosity caused by the increase in PMMA molecular weight. Furthermore, the growth rate of PVDF spherulites is in agreement with the crystallization kinetics of PVDF in their blends with PMMA shown in Figure 1B.

**Figure 4:**
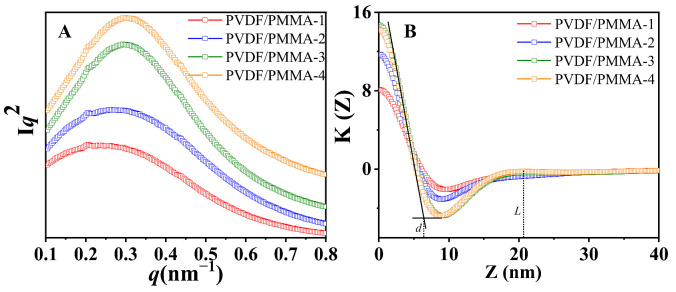
Lorentz-corrected SAXS curves (**A**) and one-dimensional correlation function curves (**B**) of PVDF/PMMA with different molecular weights of PMMA.

The necessary condition for analyzing the interlayer inclusion and interlayer exclusion structure of binary blends by SAXS is that the density difference between the two components is greater than the density difference between the crystalline and amorphous phases of each component. The densities of PVDF and PMMA are 1.78 and 1.19 g/cm^3^, respectively. The crystalline phase density (*ρ*_c_) and amorphous phase density (*ρ*_a_) of PVDF are 2.00 and 1.74 g/cm^3^, respectively. According to the calculation, the PVDF/PMMA blend system meets the requirements discussed above [33]. In order to determine the crystalline structures based on SAXS data, one-dimensional density correlation functions *K*(*z*) were calculated by the Fourier transformation of scattering curve according to Equation (1) [34]

(1) $$K\left(z\right)=[\int_{0}^{\infty }q^{2}I\left(q\right)cos\left(qz\right)dq]/2\pi \;\;\;\;\;\;\;\;\;$$

where *I* and *q* are scattering intensity and characteristic wave number, respectively. The Lorentz-corrected SAXS curves of samples are shown in Figure 4A. Only one SAXS peak can be observed in the profile of the blends containing different molecular weights of PMMA. The SAXS peak shifts to a higher *q* value with increasing *M*_w_ of PMMA in the PVDF/PMMA blends. This means that the long period of PVDF decreases because of the enhanced exclusion of PMMA chains. The obtained correlation function *K*(*z*) is shown in Figure 4B. It should be mentioned that when the crystallinity values of polymer are <50%, the average thickness readout from the correlation function can be defined as the thickness of the crystal lamellae. Hence, it is easy to obtain the average lamellar thickness (*d)*, the average long spacing (*L*), and the average thickness of the amorphous region (*AM*) for the blends and the internal crystallinity (*X*_1_ = *d*/*L*), which are listed in Table 2. The *d* and *AM* decrease with increasing molecular weight of PMMA, resulting in a smaller long period. As for the internal crystallinities (*X*_1_) calculated based on the average lamellar thickness *d* and the average long spacing, the dramatic in of amorphous thickness accounts for the bigger internal crystallinities (*X*_1_) of binary blends with increasing PMMA molecular weight.

**Figure 5:**
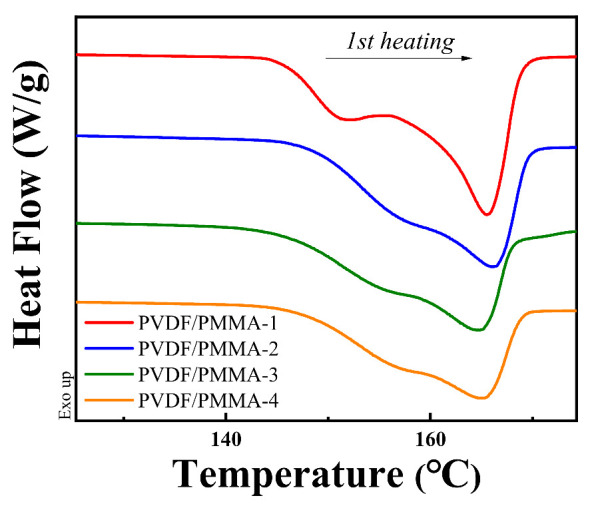
DSC curves for the first heating of PVDF/PMMA with different *M*_w_ of PMMA after complete isothermal crystallization.

**Figure 6:**
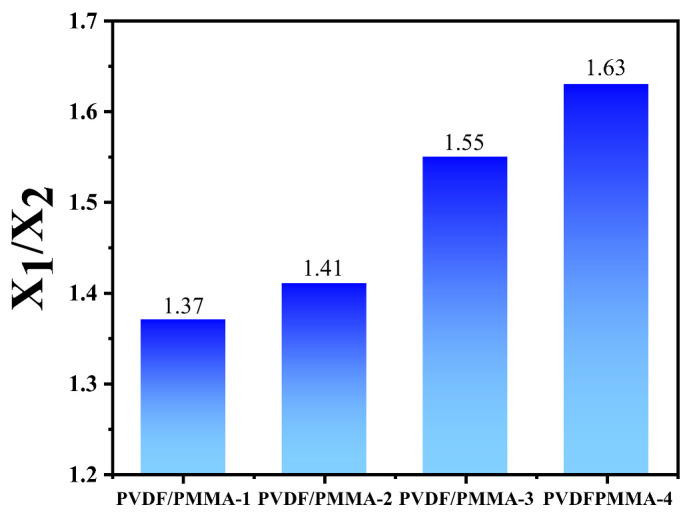
PMMA molecular weight dependences of crystallinity ratio in PVDF/PMMA blends.

The heating DSC curves of PVDF/PMMA blends with different molecular weights that were fully crystallized at 150 °C are shown in Figure 5. Due to the good miscibility between PVDF and PMMA, the melting peaks of PVDF in four specimens locate at ~165 °C, which is lower than that of pure PVDF (~175 °C). This result has good agreement with the well-known *T_m_*-depression effect [32,35]. Furthermore, the slower growth rate of PVDF resulting from the higher molecular weight of PMMA is beneficial to the formation of more perfect crystal, which leads to the closer melting peaks shown in Figure 5. This is also the reason for the lower magnitudes of *d* and *L* in Table 2. The mass crystallinity (*X_w_*) of the entire blend can be calculated by the enthalpy values:

(2) $$X_{w}=\frac{∆H_{m}}{∆H_{m}^{\theta }}\times 100\%$$

where $∆H_{m}$ and $∆H_{m}^{\theta }$ are melting enthalpy and standard melting enthalpy, respectively. The $∆H_{m}^{\theta }$ of PVDF is 104.7 J/g. It is necessary to convert the mass crystallinity (*X_w_*, from DSC) into volume crystallinity (*X*_2_). The relationship between them in the blends of PVDF and PMMA is as follows:

(3) $$\frac{1}{X_{2}}=\left(\frac{1}{\rho_{a}}+\frac{1}{\rho_{PMMA}}\right)\cdot \frac{50\%\cdot \rho_{C}}{X_{w}}-\frac{\rho_{C}}{\rho_{a}}+1$$

where $\rho_{a}$ and $\rho_{c}$ are the amorphous and crystalline phase density of PVDF, respectively; and $\rho_{PMMA}$ is the density of PMMA. The values are also shown in Table 2. Due to the higher viscosity caused by the increase in PMMA’s molecular weight, the overall crystallinity of the entire blend (*X*_2_) roughly decreases. The ratio between *X*_1_ and *X*_2_ can act as a good parameter to describe the exclusion behaviors of amorphous PMMA. The molecular weight dependences of exclusion behaviors are shown in Figure 6.

If all amorphous PMMA chains locate among PVDF lamellae, *X*_1_ and *X*_2_ should exhibit close values. On the contrary, *X*_1_ shows a higher magnitude relative to *X*_2_ when all PMMA chains are excluded from inter-fibrillar regions. In other words, lower (or higher) magnitudes of *X*_1_/*X*_2_ correspond to more (or less) PMMA locating in inter-lamellar regions. It is obvious that the molecular weight of PMMA produces remarkable effects on the ratio (Figure 6) and the exclusion behaviors of PMMA. It is observable that the higher molecular weight of PMMA results in a higher magnitude of *X*_1_/*X*_2_ in the corresponding specimens, suggesting the enhanced exclusion behavior of PMMA. The ratio for PVDF/PMMA-1 is 1.37, suggesting the co-existence of inter-lamellar and inter-fibrillar structures. With increasing molecular weight of PMMA, the ratio of *X*_1_/*X*_2_ increases monotonously. For PVDF/PMMA-4, the crystallinity ratio reaches 1.63, indicating that more PMMA chains were expelled out from the inter-lamellar regions. To understand the molecular weight of amorphous component dependence of the exclusion behaviors, the parameter of *δ* (=*D*/*G*) introduced by Keith and Padden was employed in this work. *D* and *G* are the diffusion coefficients of the amorphous component and the crystal growth rate of the crystalline component, respectively. With increasing molecular weight of PMMA, both the growth rate (*G*) of PVDF (Figure 3) and diffusion coefficient (*D*) of PMMA decrease due to the improved entanglement effect and higher viscosity. The competition between *G* and *D* determines the *δ* and exclusion of amorphous component. In this work, the decrease in *G* dominated the parameter of *δ* and the resultant exclusion behaviors of PMMA, resulting in a higher fraction of inter-fibrillar structures in the case of higher molecular weight. That is to say, if the growth rate of lamellae decreases, the chain of PMMA still has sufficient time to escape from the inter-lamellar region even when the diffusion coefficient of PMMA becomes lower because of the higher molecular weight.

**Figure 7:**
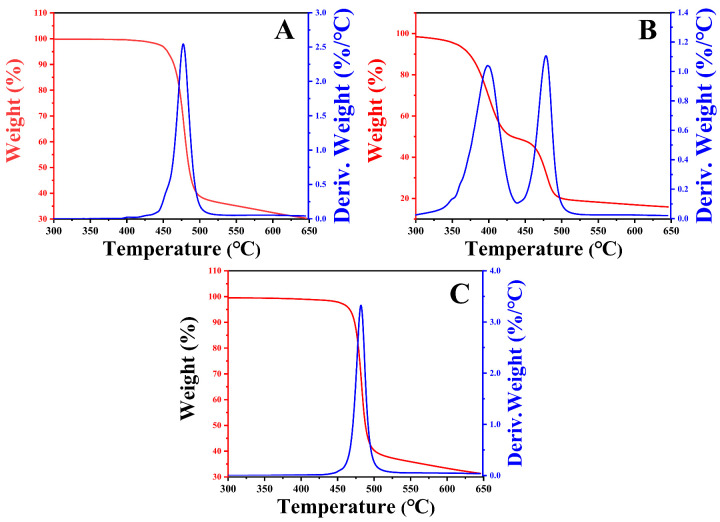
TGA curves of neat PVDF (**A**), PVDF/PMMA−1 blend before (**B**), and after (**C**) chloroform etching.

**Figure 8:**
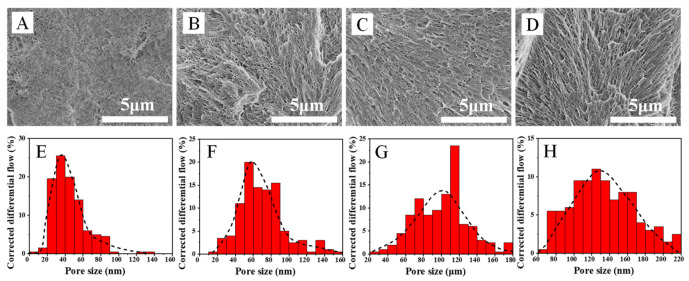
SEM images of PVDF/PMMA blends after chloroform etching with the different molecular weights of PMMA (**A**–**D**) and distribution of pore size analysis by a Nano Measurer (**E**–**H**).

**Figure 9:**
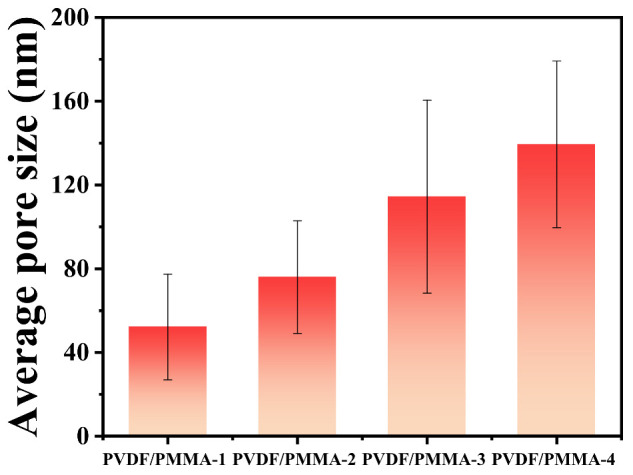
The average pore size of PVDF/PMMA blends with different molecular weights after extraction by CHCl_3_.

In our previous work, crystalline/amorphous bi-continuous structures in their miscible blends fabricated based on crystallization template have been validated by selective solvent extraction [36,37,38,39]. In this work, this strategy was employed to identify the location of PMMA in the PVDF crystal framework. Before analysis, the complete removal of PMMA-1 was evaluated by TGA. As shown in Figure 7, there are two degradation peaks of PMMA-1 and PVDF locating at ~400 and ~480 °C. Upon etching with chloroform, the former disappears, indicating the complete removal of PMMA-1. Similar results were ben obtained in other specimens (data not shown here). After extraction with chloroform (selective solvent for PMMA), the porous structures on the fracture surface correspond to the localization of PMMA in the blend. Figure 8A–D show the porous structures of four binary blends upon etching. To quantitatively analyze the effect of PMMA’s molecular weight on the morphology of the pores, the pore size on the fracture was calculated with a Nano Measurer, and the statistical pore size distribution is shown in Figure 8E–H. The resulting average pore sizes of PVDF/PMMA blends with different molecular weights after extraction are shown in Figure 9. From Figure 8 and Figure 9, our attention should be focused on the following issues: Firstly, the porous structures (from Figure 8A–D) are more evident in the specimens with high-molecular-weight PMMA, which can be attributed to the higher δ caused by lower crystal growth rate. Secondly, the average pore size increases from ~52 to ~139 nm, suggesting that more PMMA was expelled out from the inter-lamellar regions and located at the inter-fibrillar regions. Finally, the porous structures have good agreement with the crystallinity ratio (shown in Figure 6) determined by molecular weight. A higher ratio corresponds to bigger pores (Figure 8 and Figure 9) resulting from the enhanced exclusion. 

**Scheme 2:**
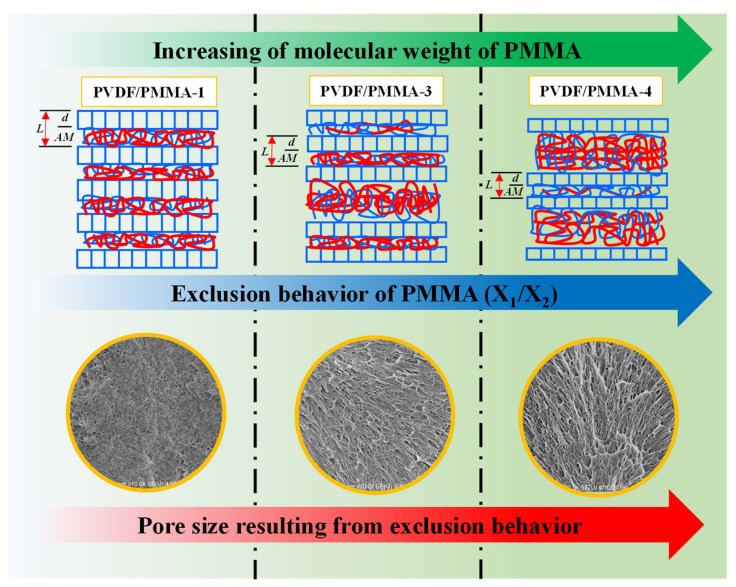
Molecular weight dependences of exclusion behaviors of PMMA in PVDF/PMMA blend.

According to the crystallinity ratio of *X*_1_ (internal crystallinity) and *X*_2_ (overall crystallinity), it is obvious that the molecular weight of PMMA produces a remarkable effect on its exclusion behaviors via varying growth rate (*G*) and diffusion coefficient (*D*). As shown in Scheme 2, for the blend of PVDF and PMMA iso-crystallized at 150 °C, more PMMA was excluded into the inter-fibrillar regions with increasing molecular weight of PMMA. The enhanced exclusion behaviors can be ascribed to the lower magnitude of the growth rate of PVDF (*G*). In this process, there is enough time for PMMA chains to diffuse and escape from the crystal growth front. As a result, PMMA enriches in inter-fibrillar regions, which is the reason for the bigger pores in PVDF upon chloroform etching.

## 4. Conclusions

In this work, PVDF/PMMA, a typical miscible crystalline/amorphous blend, was employed to investigate the effect of molecular weight of PMMA on its exclusion behaviors during the isothermal crystallization of PVDF. Our results indicate that the molecular weight of PMMA produces remarkable effects on both the growth rate of PVDF and the diffusion coefficient of PMMA. In these two parameters, a decrease in *G* dominates the parameter of *δ* and the resultant exclusion behaviors of PMMA, resulting in a higher fraction of inter-fibrillar structures in the case of higher molecular weight. The localization of PMMA was validated by the porous structures after the extraction of PMMA by chloroform. With increasing molecular weight of PMMA, more PMMA is excluded from the inter-lamellar region, resulting in bigger pores. Our results are significant not only for the basic understanding of crystallization in polymer blends but also for the fabrication and structure control of porous structures based on crystal templates.

## Figures and Tables

**Table 1:** PMMA with different molecular weights used in this work.

Samples	PMMA-1	PMMA-2	PMMA-3	PMMA-4
*M*_w_ (g/mol)	15,000	120,000	350,000	996,000
PDI	1.58	1.71	1.90	2.26

**Table 2:** The lamellar thickness *d*, the long spacing *L*, thickness of the amorphous region *AM* and the crystallinity for the blends at 150 °C.

Samples	d (nm)	L (nm)	AM (nm)	*X*_1_ (SAXS)	*X*_2_ (DSC)	*X*_1_/*X*_2_
PMMA-1	7.33	25.86	18.53	28.3%	20.7%	1.37
PMMA-2	6.81	22.93	16.12	29.7%	21.1%	1.41
PMMA-3	6.65	21.37	14.68	31.1%	20.1%	1.55
PMMA-4	6.43	20.60	14.18	31.2%	19.2%	1.63

## Data Availability

Not applicable.
